# Association Study of rs1333040 and rs1004638 Polymorphisms in the 9p21 Locus with Coronary Artery Disease in Southwest of Iran

**DOI:** 10.7508/ibj.2016.02.008

**Published:** 2016-04

**Authors:** Khadijeh Golabgir Khademi, Ali Mohammad Foroughmand, Hamid Galehdari, Saied Yazdankhah, Mahdi Pourmahdi Borujeni, Zahra Shahbazi, Parvaneh Dinarvand

**Affiliations:** 1 Dept. of Genetics, Faculty of Science, Shahid Chamran University of Ahvaz, Ahvaz, Iran;; 2 Dept. of Medicine, Jondi Shapour University of Medical Science, Ahvaz, Iran;; 3 Dept. of Food Hygiene, Faculty of Veterinary medicine, Shahid Chamran University of Ahvaz, Ahvaz, Iran

**Keywords:** Coronary artery disease, Single nucleotide polymorphisms, Genetic association study, Iran

## Abstract

**Background:**

Coronary artery disease (CAD) is a multifactorial and heterogenic disease. Recently, genome-wide association studies have reported that rs1333040 (C/T) and rs1004638 (A/T) single nucleotide polymorphisms (SNPs) in the 9p21 locus have very strong association with CAD. This study aimed to examine these associations in Southwest of Iran.

**Methods:**

Blood samples were collected from 200 CAD patients and 110 healthy individuals with no CAD. The association of two SNPs with CAD was evaluated by PCR and restriction fragment length polymorphism.

**Results:**

Chi-square test showed no association between rs1333040 SNP and CAD (X^2^: 4.66, df: 2, *P*=0.09). Also, there was no association between rs1004638 SNP and CAD (X^2^: 0.27, df: 2, *P*=0.88).

**Conclusion:**

No association was observed between rs1333040 and rs1004638 SNPs in the 9P21 region and CAD in Southwest of Iran.

## INTRODUCTION

Coronary artery disease (CAD) and myocardial infarction (MI) are the leading causes of mortality worldwide^[^^1^^]^. During CAD, athero-sclerosis plaques build up in the interval walls of coronary arteries and gradually block them. This event limits the blood flow to myocardium and leads to significant problems^[^^2^^]^. Unlike uncommon single gene disorders, 80% of death worldwide is caused by 20 diseases. Cancer, infections, and CAD are responsible for many of these deaths^[^^3^^]^. CAD is a multifactorial and heterogenic disease that is caused by both genetic and environmental factors, as well as by their interaction with each other^[^^4^^,^^5^^]^.

Genetic factor accounts for 50% of the susceptibility to CAD^[^^6^^]^. Other cardiovascular risk factors such as hypertension, abnormal lipid metabolism, smoking, and diabetes are responsible for 50% to 60% of the susceptibility to CAD and ischemic stroke. Modifying some of these risk factors such as smoking, hyper-tension, and blood cholesterol levels has confirmed that 30% to 40% of deaths worldwide from CAD can be prevented ^[^^3^^,^^7^^]^

Many candidate genes have been suggested to be associated with CAD, MI, and many other polygenic diseases such as hypertension and diabetes^[^^3^^]^. Genome-wide association studies have reported that single nucleotide polymorphisms (SNPs) in the 9p21 locus have a very strong association with CAD and MI^[^^8^^-^^10^^]^. The association between SNPs in the 9p21 locus and CAD or MI has been confirmed in many populations, including Caucasian, Chinese, Korean, Italian, and Japanese^[^^1^^,^^8^^,^^11^^,^^12^^]^. To date, Korean, Indian, Japanese, Chinese, and Pakistani have been the only Asian populations studied for SNPs at the 9p21 region^[^^1^^,^^11^^-^^13^^]^. The 9p21 region has also shown to be associated with ischemic stroke, type 2 diabetes, and abdominal aortic aneurysm^[^^14^^-^^16^^]^. 

The 9p21 locus is a 58,000-bp region containing CDKN2B-AS (cyclin-dependent kinase inhibitor 2B antisense RNA), which encodes an antisense non-coding RNA ^[^^17^^]^. CDKN2B-AS is located next to the CDK inhibitor genes, namely CDKN2A (cyclin-dependent kinase inhibitor 2A) and CDKN2B, which both inhibit CDK4 and regulate the cell growth. In reality, the sequence of CDKN2B-AS overlaps that of CDKN2B^[^^18^^]^.

The cell growth is regulated by two pathways. In one pathway, retinoma protein inhibits cell growth in the G1 phase and in the other one, p53 inhibits cell growth in the G1 and G2 phases. CDNK2A or CDKN2B proteins can arrest the retinoma protein pathway and stop cells in the G1 phase. Also, an alternative transcript of CDKN2A arrests both pathways^[^^19^^]^.

The 9p21 region contains SNPs that accounts for the increased risk of CAD. In addition, the SNPs at CDKN2A or CDKN2B genes show a weak association with CAD. The association between CDKN2B-AS region and atherosclerosis may be related to the antiproliferative action of the CDNK2A or CDKN2B genes^[^^20^^]^. This region has been the target of cancer studies because of genes (CDKN2A and CDKN2B) that regulate cell growth. The CDKN2A and CDKN2B products inhibit smooth muscle proliferation in the vascular endothelium. The polymorphisms at the 9p21 region may induce the higher expression of the CDKN2B-AS transcript and then inhibit the expression of the CDKN2A and CDKN2B genes. Moreover, multiple conserved enhancers in the 9p21 locus have shown to be associated with the up-regulation of some genes that induce proliferation^[^^20^^]^.

Recently, genome-wide association studies have reported two SNPs (rs1333040 and rs1004638) at the 9p21 chromosome that were associated with CAD and MI^[^^5^^,^^7^^,^^21^^]^. Thus, we carried out a case-control association study between rs1333040 (C/T) and rs1004638 (A/T) polymorphisms with CAD in Southwest of Iran.

## MATERIALS AND METHODS

**Study subjects**

The present study was performed on 200 unrelated cases (26-65 years of age) and 110 unrelated controls (48-86 years of age). The cases were randomly selected from the individuals referred to Angiography Department of Golestan and Emam Khomeini Hospitals in Ahvaz, Khuzestan Province, Iran. CAD patients had ≥70% luminal narrowing in a major epicardial artery by coronary angiography, whereas the controls had no detectable stenosis by angiography and were generally in a good health. Blood samples were collected from the case and control groups. Moreover, at the time of blood sampling, a complete clinical history including CAD risk factors such as gender, age, history of hypertension, triglyceride level, and history of diabetes mellitus was collected from both groups. The demographic details for the case and control subjects are shown in Table 1. These risk factors were ascertained based on the diagnosis of a clinician and relevant medical records of medication and laboratory tests. Hypertension was defined as systolic blood pressure of >140 mm Hg or diastolic blood pressure >90 mm Hg. Diabetes was determined as ongoing therapy of diabetes or fasting blood sugar of >126 mg/dL. Hyper triglyceride level was defined as a fasting triglyceride level of 150 mg/dL or higher.

**PCR analysis**

**Fig. 1:**
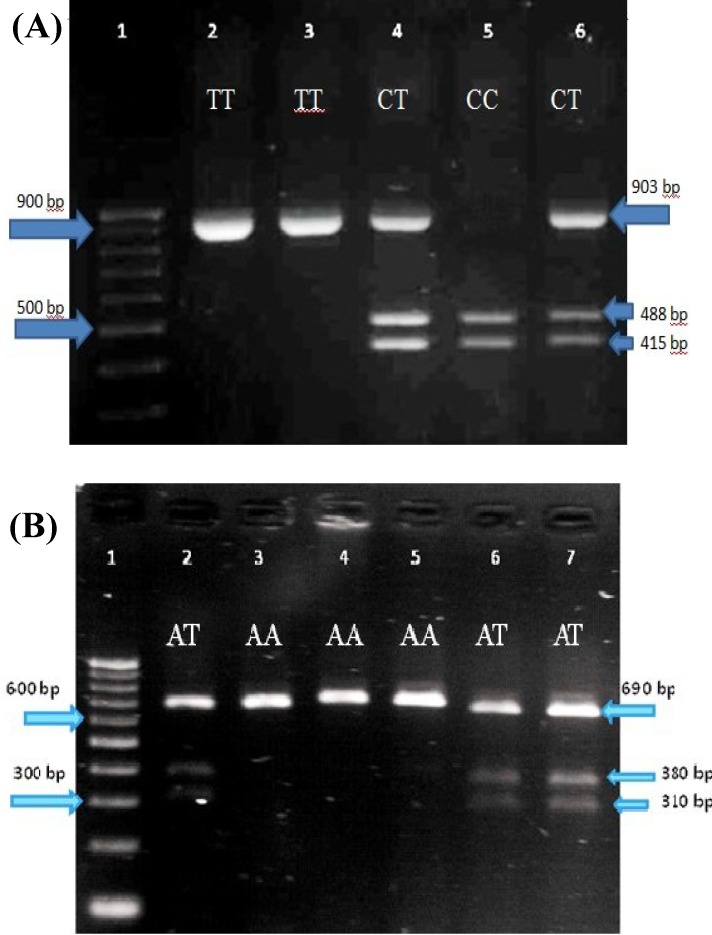
Restriction fragment length polymorphism for (A) rs1333040 and (B) rs1004638

Human genomics DNA was extracted from blood samples using the Diatom DNA prep 100 extraction kit (Cinnagen, Iran). Genotyping of the investigated SNPs (rs1333040 and rs1004638) was analyzed by using the PCR and restriction fragment length polymorphism. The two studied SNPs were 32185 nucleotides away from each other and should be amplified by two pairs of PCR primers. The oligonucleotide primers 1333040F (5′AAGAAGGCTGGTAGCAGGAAG3′), rs1333040R (5′ATCACCACCCAAACACCAGT3′), rs1004638F (5′CATGCTTAGCTGAAGGGATCCGA 3′), and rs1004638R (5′GAAGACTGGGGAAGGTG TCC3′) used in PCR amplification were designed using Primer3 (version 1.0). The PCR reaction was carried out in a final reaction volume of 25 µ l using 3 µl genomic DNA as template. Reaction mixture (rs1004638) contained 2 µl each primer, 0.3 µl Taq DNA polymerase (Gen Fanavaran, Iran), 2.5 µl 10× buffer, 0.5 µl dNTP, and 0.8 µl MgCl_2_. The master mix composition (rs1333040) was consisted of 2 µl each primers and 12.5 µl Taq DNA Polymerase Master Mix Red (Ampliqon, Denmark). The cycling program was as: an initial denaturation at 95ºC for 5 minutes, followed by 35 cycles of denaturation at 95ºC for 30 seconds, annealing at 63ºC for 35 seconds (rs1004638) and at 68ºC for 30 seconds (rs1333040) and extension at 72ºC for 45 seconds (rs1004638) and 60 seconds (rs1333040). The amplification was terminated after 5 minutes of extension at 72ºC. The sizes of PCR products for rs1333040 and rs1004638 polymorphisms were 900 and 690 bp, respectively. Then the PCR products of two SNPs were digested with two restriction endonucleases (Vivantis, Malaysia), named *BsmI* for rs1333040 (Fig. 1A) and *AflII* for rs1004638 (Fig. 1B). Digestion product was detected by agarose gel electrophoresis.

**Table 1:** Demographic characteristics of the participants in each group

**Variables**	**Cases**	**Controls**
Age (y)	56.30 ± 9.06	57.67 ± 9.38
Sex		
Male	114	51
Female	86	59
Blood pressure		
Affected	55	22
Not affected	145	88
Diabetes mellitus		
Affected	48	9
Not affected	152	101
hypertriglyceridemia		
Affected	61	14
Not affected	139	96

Data are presented as mean ± SD or frequency.

**Statistical analysis**

Statistical analysis was performed by SPSS version 16.0 (SPSS Inc., Chicago, IL, USA). The differences of the allelic and genotype frequencies between the case and control groups were tested for Hardy-Weinberg equilibrium using a chi-square (X^2^) test with one degree of freedom. The association between the genotypes and CAD as well as between the clinical covariates (such as gender, history of hypertension, triglyceride level, and diabetes mellitus) and CAD were analyzed by logistic regression analysis. A *P* value of 0.05 or less was considered to be statistically significant.

## RESULTS

The logistic regression analysis and X^2^ test were performed for all variables. Among all of the risk factors, only diabetes and hypertriglyceridemia showed a very strong association with CAD in the present study (*P*≤0.05). We carried out a standard genotype analysis for two SNPs, which were selected based on the strength of association in previous studies. The odds ratios were determined for rs1004638 and rs1333040 genotypes. No association of two SNPs in the cases and controls with CAD were observed in the present study even after adjustment for the clinical covariates of age, gender, diabetes, and hypertension (*P*>0.05) (Table 2). 

The X^2 ^test did not confirmed the association between the genotypes of two SNPs and CAD in the cases and controls. The results for rs1004638 and rs1333040 were as follows: X^2^: 0.27, df: 2, *P*=0.88 and X^2^: 4.66, df: 2, *P*=0.09, respectively. In these two SNPs, there were no significant differences in genotype frequencies between men and women in the case or in the control groups. The results of sex calculation for rs1333040 and rs1004638 were as follows: X^2^: 0.29, df: 2, *P*=0.87 and X^2^: 0.02, df: 1, *P*=0.89, respectively. 

## DISCUSSION

Several recent genome-wide association studies have reported the association of two SNPs, namely, rs1333040 and rs1004638, in the 9p21 region with CAD. The association of these SNPs with CAD has been confirmed in German, North Indian, and Chinese Han populations^[^^5^^,^^7^^,^^21^^]^. Therefore, in the present study, we investigated the association of two SNPs (1333040 and 1004638) with CAD in Southwest of Iran. The results of our study were not in accord with the results of previous studies, which showed that there is no significant association between these SNPs and CAD. 

CAD is a multifactorial and heterogenic disease that is produced by both genetic and environmental factors. One of the difficulties of studying CAD is finding matched cases and controls in terms of age. CAD occurs in older ages; therefore, it is possible that controls are affected in the future. To minimize this effect, only controls over 48 were studied among the individuals with normal angiographic result. On the other hand, the effect of environmental factors becomes more prominent with age. Thus, many elderly individuals were excluded from the present study because it was not clear whether the cause of CAD was genetically or environmentally. For this purpose, the case and control groups were selected from those who were ≤65 and ≥48 years, respectively. Cases included those who had afflicted by this disease in younger ages and had high disease susceptibility. Controls had no CAD in spite of aging and therefore, might have the ability to resist CAD. With these criteria, approximately 310 samples from 1290 patients met the eligibility criteria and were recruited in our study. 

**Table 2:** Association analysis of variables with CAD

**Variables**		**Alleles**			**Genotypes**			**OR (95% CI)** **unadjusted**	***P*** **value**	**OR(95% CI)** * **adjusted**	***P*** **value**
		**Case ** **(%)**	**Control ** **(%)**		**Case ** **n=200**	**Control** **n=110**					
rs1333040		C: 0.27	C: 0.25		CC:5	CC:3		1		1	
					CT:98	CT:50		1.16 (0.27-5.07)	0.84	1.08 (0.24-4.92)	0.76
		T: 0.73	T: 0.75		TT:97	TT:57		1.03 (0.24-4.48)	0.97	1.27 (0.28-5.79)	0.92
rs1004638		A: 0.85	A: 0.81		AA:139	AA:69		1		1	
					AT:61	AT:41		0.78 (0.47-1.27)	0.32	0.76 (0.45-1.29)	0.31
		T: 0.15	T: 0.19		TT:0	TT:0					
Hypertension								1.52 (0.87-2.66)	0.15		
Diabetes Mellitus								3.54 (1.67-7.54)	0.001		
Gender								1.53 (0.96-2.45)	0.70		
hypertriglyceridemia								3.01 (1.59-5.69)	0.001		

SNPs: Single Nucleotide Polymorphisms, OR: Odds Ratio, CI: Confidence Interval,

* OR: adjusted Odds Ratio according to the age, gender, and histories of DM and hypertension; *P*≤0.05 is significant

Another difficulty in the present study was matching participants for gender. Because of the protective role of estrogen, more men were diagnosed with CAD compared to women. A non-modifiable risk factor for CAD is male gender. In this study, the number of female and male subjects was approximately equal. Also, the chance of developing CAD were found to be 1.53 times more in men than in women, which was not statistically significant. Among the modifiable risk factors, Diabetes Mellitus and hypertriglyceridemia have been shown to have a strong association with CAD. Hypertension is another factor that did not show any statistically significant association with CAD.

The inclusion and exclusion criteria in this study were sex and age, and in both groups, angiography confirmed the presence or absence of stenosis because angiography has the highest accuracy among the available methods. In analysis of the genotypes of the samples, in rs1333040 polymorphism, the frequency of CT genotype was found to be higher in patients in comparison with controls. The frequency of C and T alleles was also higher in patients and the healthy group, respectively. Overall, this polymorphism was not significantly associated with CAD. In rs1004638 polymorphism, the frequency of both AA and AT genotypes was higher in patients and healthy individuals, respectively. In addition, the A allele frequency in patients and T allele frequency in healthy individuals were higher. This polymorphism also showed no association with CAD.

The sample size is an important factor in association studies. In the present study, the limited numbers of samples were due to a large number of eligibility criteria that should be met in our sampling. However, the strong point of this study was the application of angiography for the accurate selection of the samples.

Various phenotypes consisting of abdominal aortic aneurysm, ischemic stroke, intracranial aneurysm, type 2 diabetes mellitus, osteoporosis, possibly Alzheimer's disease, and CAD have shown to have an association with the 9p21 locus. The observed association of CAD and the 9p21 region could be mediated by means of complex mechanisms of atherosclerosis. More mechanistic studies are essential to define the potential of SNPs at the 9p21 region on plaque instability and intra-arterial thrombosis^[^^8^^]^. Also, future case-control studies based on a large sample size are required to perform and evaluate the functional role of the 9p21 locus in the etiopathology of heart disease.
